# Diagnosing moyamoya syndrome using ultrasound - a case report

**DOI:** 10.1186/s12883-015-0518-7

**Published:** 2015-12-22

**Authors:** Tobias Braun, Martin Juenemann, Dursun Gündüz, Stefanie Schmetsdorf, Florian Roessler, Astrid Grams, Carolin Gramsch, Christian Tanislav

**Affiliations:** Department of Neurology, Justus Liebig University, Klinikstrasse 33, 35392 Giessen, Germany; Department of Cardiology and Angiology, Justus Liebig University, Giessen, Germany; Department of Neuroradiology, Justus Liebig University, Giessen, Germany; Department of Neuroradiology, University Innsbruck, Innsbruck, Germany

**Keywords:** Moyamoya syndrome, Moyamoya disease, Stroke, Intracerebral artery, Risk factors

## Abstract

**Background:**

Moyamoya syndrome is a vasculopathy characterised by progressive occlusion of the cerebral arteries resulting in the development of abnormal collateral circulation. To diagnose this syndrome, imaging of the cerebral arteries is required including CT- or MR-angiography and conventional angiography. We present a case of moyamoya disease with typical findings detected in the sonography. The diagnosis was suspected after reviewing the initial ultrasound images of the cerebral arteries with evidence for obliterated intracranial arteries and the detection of an existing collateral circulation network.

**Case presentation:**

A 62 years old male patient presented in the hospital’s emergency department with symptoms indicating a subacute cerebrovascular event. Immediate sonographic studies showed a right-sided pulsatile Doppler-signal in the common and internal carotid arteries, suggestive of distal stenoses. In addition, the transcranial examination indicated obliteration of both middle cerebral arteries. Numerous arterial vessels suggestive of leptomeningeal collateral arteries revealed a strong arterial leptomeningeal flow. At this stage of the diagnostic work-up, the collateral circulation network, characteristic of moyamoya disease, was indicated by sonography. Moyamoya syndrome was verified by conventional angiography. The aetiological work remained empty, so the diagnosis of moyamoya disease was established.

**Conclusion:**

Our case report indicates that sonography can be a useful tool for detecting the vaculopathy in moyamoya syndrome. In case routine procedures, such as the CT- or MR-angiography, with evidence for obliterated intracerebral arteries, ultrasound studies might provide important information regarding an existing collateral network in the scope of a moyamoya syndrome.

## Background

A moyamoya syndrome is an occlusive disease of cerebral arteries, mainly found in the circle of Willis. This disease is characterized by an abnormal collateral circulation network at the base of the brain that looks like a “puff of smoke,” or “moyamoya” in Japanese [1–3]. The clinical presentation includes various neurological manifestations such as strokes, seizures or mental retardation, mainly caused by brain ischemia and cerebral bleeding [1]. While computed tomography and MRI-angiography are valid diagnostic tools, the best procedure for visualizing typical findings in moyamoya syndrome is conventional angiography [4]. In a recent case we found evidence supporting the use of ultrasound screenings as a valid method to identify a vasculopathy in the scope of a moyamoya syndrome prior to the comprehensive testing for full diagnosis. Our observation is of particular interest, as the transcranial Doppler/duplex sonography, established as a procedure to examine the main branches of the brain arteries, might also be suitable to investigate smaller intracranial vessels, as demonstrated in our patient with moyamoya vasculopathy. The wide availability of the method and the low risk for unfavourable procedural outcomes are in this context of high relevance. For the case presentation the patient gave written informed candent.

## Case presentation

A 62-year-old male presented to the hospital’s emergency department with right-sided mild hemiataxia and mild hemiparesis. Fourteen days prior to admission, the patient had noticed numbness of the fourth and fifth finger of the right hand, which continued to spread up the entire arm. The medical history included hypertension with a prescription of ramipril 5 mg daily.

Initial physical examination revealed a mild hemiparesis with hemiataxia and choreiform movements of the right leg. His left extremities showed increased reflexes-levels as well as a right-sided positive Babinski sign. Otherwise he was in good medical condition. Blood pressure was 140/90 mmHg.

**Fig. 1:**
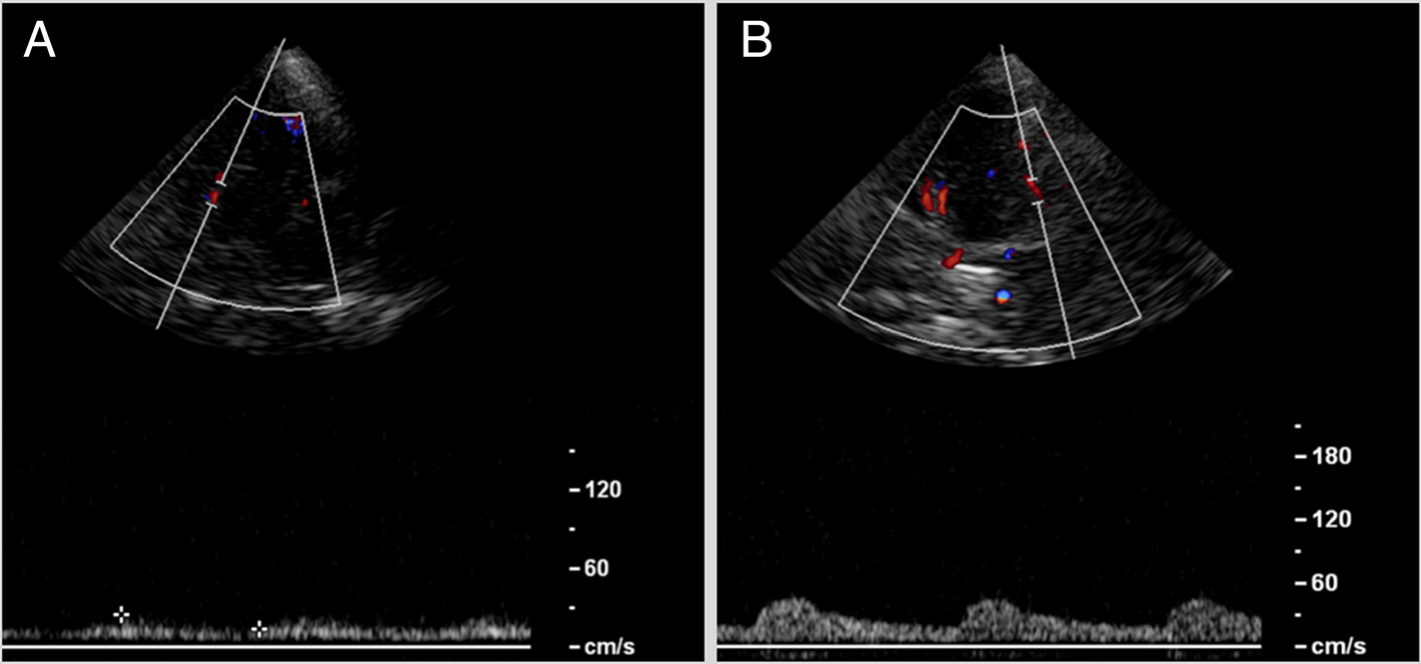
Middle cerebral artery left (**a**); due to obliteration the Doppler-spectrum is low pulsatile, the corresponding detected flow velocities are also low. In contrast flow velocities detected in collaterals are higher (**b**)

**Fig. 2:**
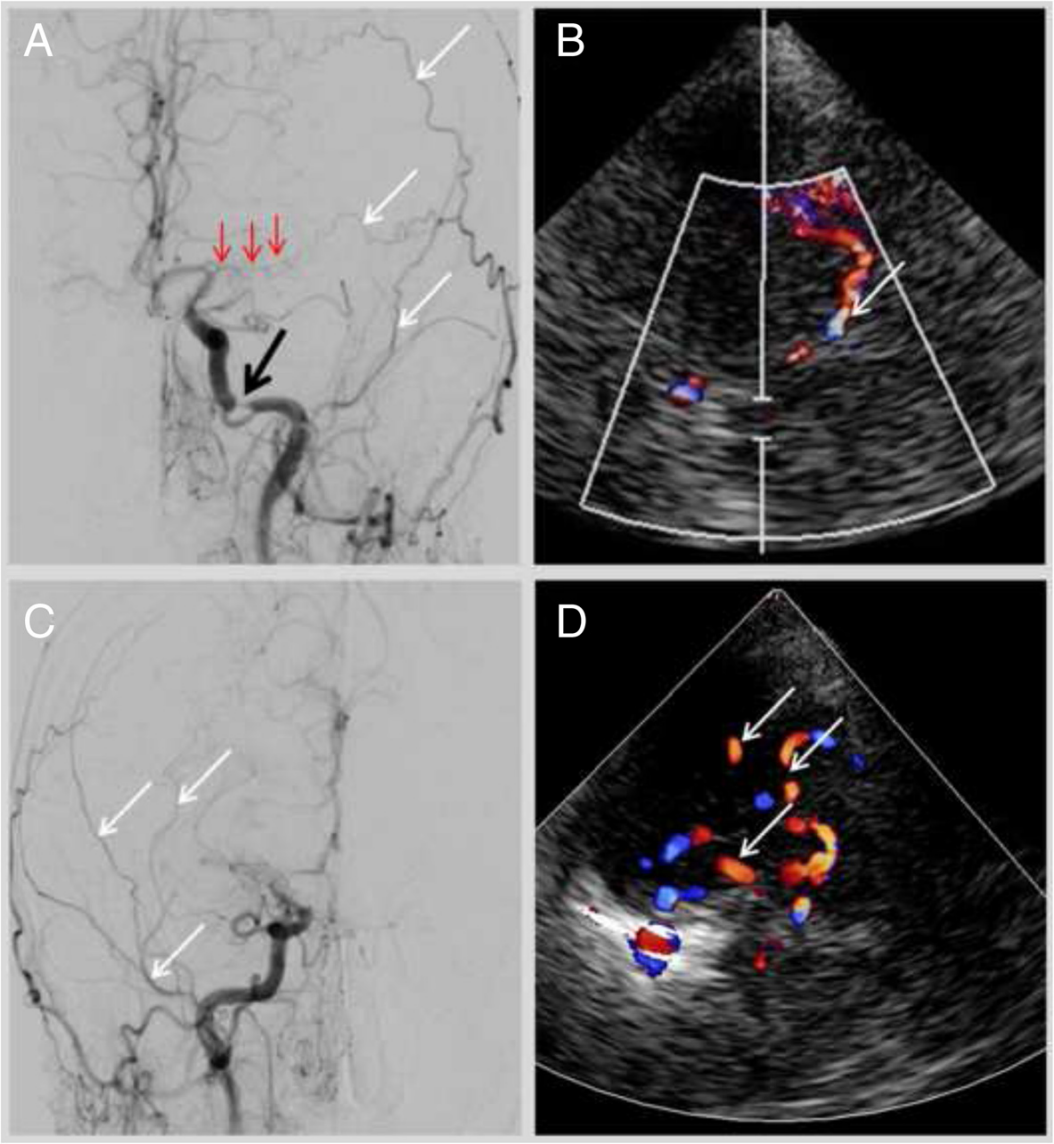
Cerebral digital subtraction angiography left (**a**) in frontal projection. Severe stenosis of the carotid artery (*black arrow*); the middle cerebral artery is obliterated (*red arrows*). Branches of the collateral network with leptomeningeal anastomoses are indicated by white arrows. Image **b** demonstrates a leptomeningeal collateral branch in the duplex-sonography. The digital subtraction angiography right (**c**) indicates collateral vessels (*white arrows*) by obliterated middle and anterior cerebral artery; correspondently the transcranial duplex-sonography right (**d**) demonstrates numerous arterial vessels assembling the collateral network

**Fig. 3:**
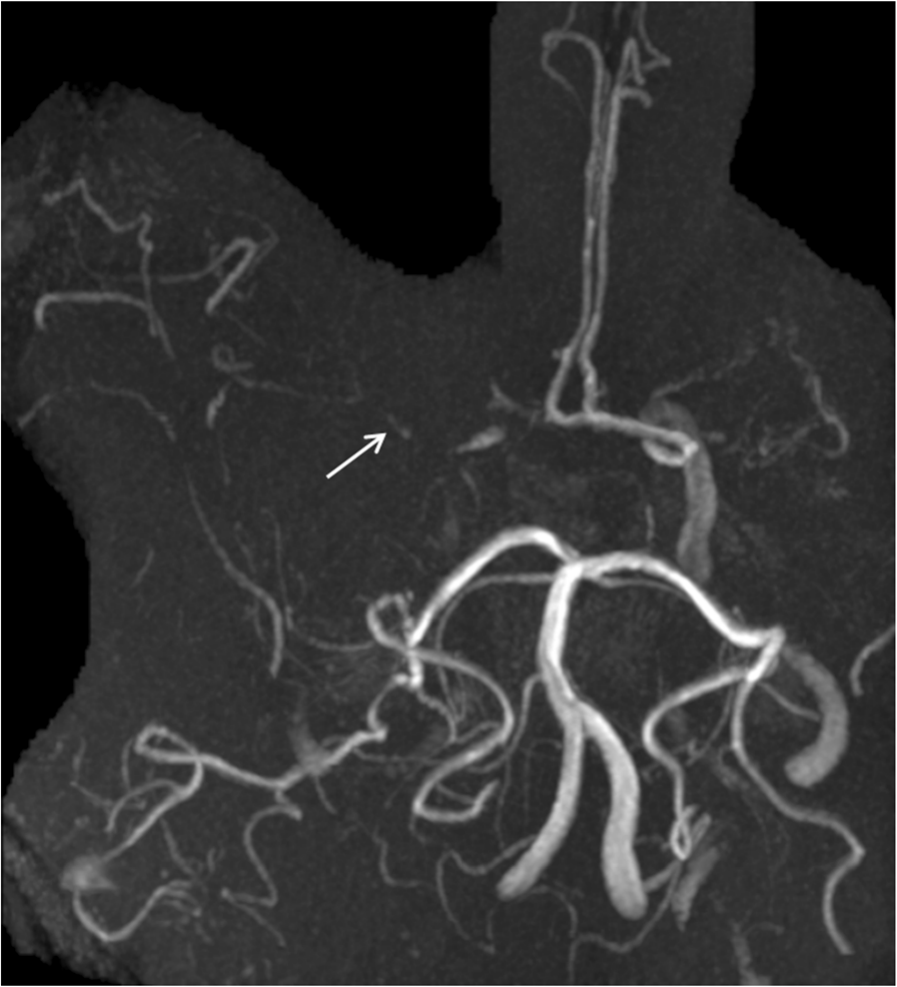
Cerebral MR angiography indicates the obliteration pathology in the anterior circulation; the middle cerebral arteries are bilaterally rudimentary visible (*arrows*)

**Fig. 4:**
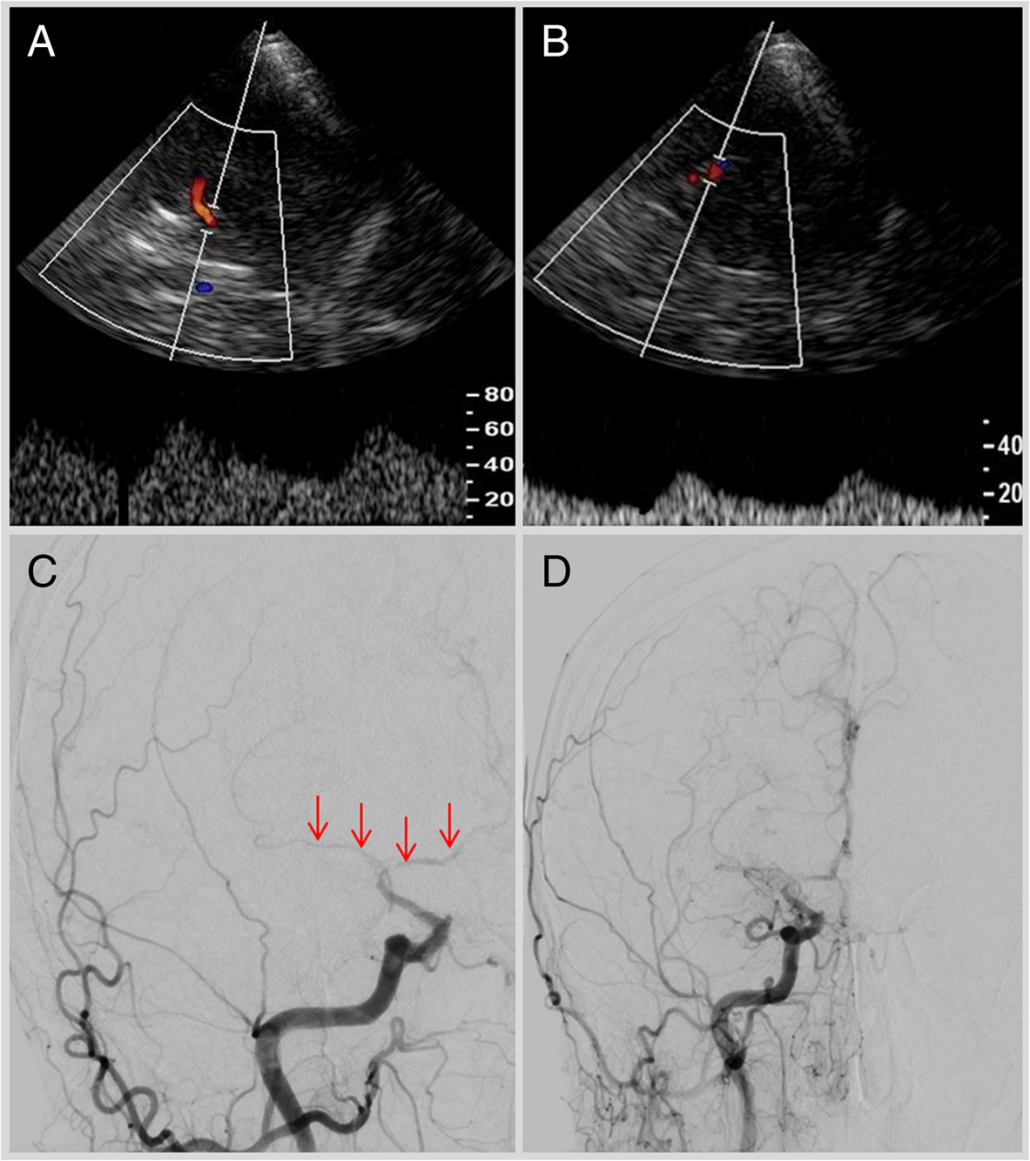
Transcranial Doppler- and duplex-sonography right, showing deteriorated Doppler-spectrum in the distal portion of the internal carotid artery (**a**) and proximal portion of the middle cerebral artery (**b**) indicating the vessel obliteration; in accordance with sonographic findings the conventional angiography (**c**) confirmed the obliteration of the middle cerebral artery and anterior cerebral artery (*red arrows*). Image (**d**) demonstrates the collateral network

Initial sonographic studies showed a right-sided pulsatile Doppler-signal in the common and internal carotid arteries, suggestive of distal stenosis. In addition, there was moderate increase in flow velocities in the basilar-artery. In contrast the Doppler-spectrum in the intracranial vessels indicated widespread obliteration. Numerous arterial vessels suggestive of leptomeningeal and lenticulostriate collateral arteries revealed a strong collateral flow (Figs. 1 and 2). These findings were interpreted as evidence of moyamoya-syndrome. MRI-angiography (3 Tesla) also suggested the suspected disorder; both middle cerebral arteries were obliterated (Fig. 3). The diagnosis was eventually verified using conventional angiography, which revealed severe stenoses of the internal carotid arteries in the siphon portion, the obliterated vessels of the circle of Willis and the extended collateral leptomeningeal and lenticulostriate network (Figs. 2 and 4).

**Fig. 5:**
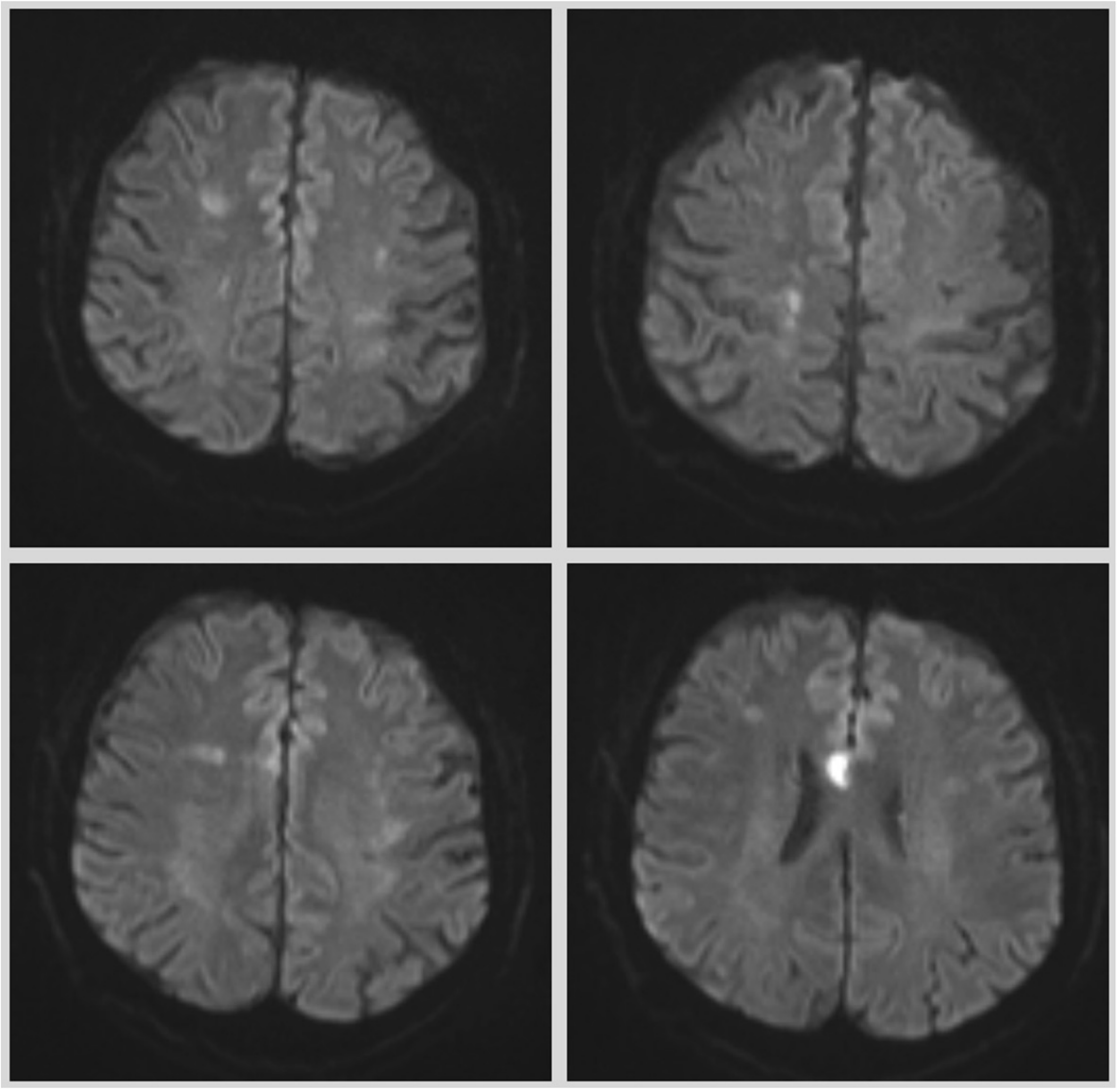
Brain MRI, diffusion weighted imaging showing multiple bilateral acute ischaemia

Further diagnostic including brain imaging revealed the disease burden caused by the vasculopathy. A hypodense lesion dorsolateral to the left-sided caudate nucleus, measuring 15 × 7mm, was detected in the CT scan. Magnetic resonance imaging (diffusion weighted imaging on MRI) showed bilateral water shed ischemic lesions at various stages of development suggesting a haemodynamic infarction mechanism (Fig. 5). MRI findings also indicated previous old haemorrhage in the left-sided putamen.

A cardiac workup revealed a persistent foramen ovale without any indication for deep venous thrombosis or pulmonary embolism. Holter-ECG provided no evidence for an arrhythmia such as atrial fibrillation. An etiological workup ruled out autoimmune disorders and the cerebrospinal fluid showed no abnormalities. As no cause was found, the patient was diagnosed with moyamoya disease.

The patient was prescribed a medication with aspirin and simvastatin. Due to recurrent symptoms during hospitalization (dysarthria, worsening of hemiparesis) clopidogrel was added to the treatment plan for 3 months. At discharge, the patient only suffered from mild hemiparesis. On subsequent follow-up, the vascular status did not change and clinically the patient recovered entirely. After 3 months, the secondary prevention treatment plan was reduced to aspirin as single therapy.

## Discussion

In the current medical practice, as soon as the diagnosis of moyamoya syndrome is suspected, conventional angiography is considered as most suitable for detecting typical findings and confirming the diagnosis [4, 5]. Therefore findings detected in previous examinations are relevant to justify the invasive procedure of conventional angiography. In our presented case, the transcranial ultrasonography gave the first indication of moyamoya syndrome. Arterial vessels were found using Doppler/duplex ultrasound unveiling the arterial collateral network and revealed the obliterated middle cerebral artery. The diagnosis was suspected prior to the detection of specific findings in CT or MRI-angiography.

Moyamoya syndrome is a rare cerebrovascular disorder characterised by progressive occlusion of the internal carotid arteries and their branches [5]. In some cases, it also affects the extra-cranial portions of the internal carotid artery. The so-called bottleneck sign, a narrowing of the internal carotid artery above the bulb, represents a typical vascular finding in moyamoya disease [6]. In our patient this finding was not depicted, indicating, at the stage the vasculopathy was diagnosed, the disorder affected mainly the intracranial portions of the brain arteries. In the course of the disease the vessel occlusion leads to the formation of collateral circulation networks needed to sustain sufficient perfusion of the brain. As a result, patients are predisposed to ischemic and haemorrhagic events. Patients with moyamoya syndrome usually present therefore with stroke due to ischemia or intracranial bleeding, but they also complain of migraine-like headache (due to dilated meningeal and leptomeningeal collaterals) or choreiform movements (due to dilated lenticulostriatal moyamoya vessels in the basal ganglia) [7, 8]. The actual cerebrovascular event in our patient was an ischemic stroke; the brain imaging indicated an old intracranial haemorrhage. Within the stroke workup the sonography of the brain arteries detected in an early diagnostic step the characteristic moyamoya sign, the branches of a collateral network. The indication of moyamoya syndrome was therefore evident far in advance standard procedures for imaging intracranial vessels were performed.

The results of the etiological workup are frequently inconclusive and the cause of the disease remains unclear [1, 8–12]. Idiopathic moyamoya syndrome is the more common type of the disease and is often referred to as true moyamoya disease. It is most prevalent in Asia, (Incidence in Japan: 0.94 in 100.000) while in the Unites States the incidence is significantly lower (0.094 in 100.000) [1, 13–15]. Studies have shown that in Caucasian patients the female sex predominates (ratio of 4.25:1) with a more benign course of the disease [1]. The secondary type, simply called moyamoya syndrome, is associated with other disorders, such as sickle cell diseases, graves’ disease, cranial irradiation and other causes [2, 8]. Our patient’s medical history and an extensive workup did not reveal any suspicious findings. As a result, the diagnosis of moyamoya syndrome without a causative disorder was concluded.

It is important to establish the diagnosis of moyamoya syndrome because it determines the acute and subsequent therapeutic management. When an underlying pathology is proved, therapeutic management is guided by treatment of this disorder. Evidence regarding the efficacy of various treatment modalities for moyamoya syndrome is limited. In case of stable clinical conditions, the conservative management may be the preferable option. Further treatment measures follow the current recommendations in stroke therapy. Platelet aggregation inhibitors and other measures such as blood pressure control might be considered to prevent cerebrovascular events [5, 16]. In case of unstable haemodynamic conditions surgery (bypass surgery) may also be considered. Interventional treatment (angioplasty or stenting) was not proven to be successful in treatment of moyamoya disease; it might be an option only in some selected cases [5]. In our patient we succeed with a conservative management, the patients’ clinical status remains stable with minor deficits irrelevant for coping with daily tasks.

## Conclusion

Our case report indicates that extra- and intracranial Doppler/duplex Sonography could be an important tool in the diagnostic algorithm for diagnosing a moyamoya syndrome. Numerous vessels suggestive of leptomeningeal and lenticulostriate collaterals were obvious, guiding the diagnosis of moyamoya syndrome. Additional specific findings such as obliterated middle cerebral arteries in the CT- or MRI-angiography were detected later. Ultrasound studies may be therefore helpful to enforce the suspected diagnosis of a vascular disorder in the scope of moyamoya syndrome. Sonography should be further investigated with larger cohorts in the diagnostic evaluation of moyamoya to determine its diagnostic accuracy.

## Consent

Written informed consent was obtained from the patient for publication of this case report and any accompanying images. A copy of the written consent is available for review by the corresponding author.

## Abbreviations

CT: computed tomography
ECG: electrocardiogram
MRI: magnet resonance imaging

## References

[1] Burke GM, Burke AM, Sherma AK, Hurley MC, Batjer HH, Bendok BR (2009). Moyamoya disease: a summary. Neurosurg Focus.

[2] Houkin K, Ito M, Sugiyama T, Shichinohe H, Nakayama N, Kazumata K (2012). Review of past research and current concepts on the etiology of moyamoya disease. Neurol Med Chir (Tokyo).

[3] Kuroda S, Houkin K (2008). Moyamoya disease: current concepts and future perspectives. Lancet Neurol.

[4] Liu W, Xu G, Liu X (2013). Neuroimaging diagnosis and the collateral circulation in moyamoya disease. Interv Neurol.

[5] Kronenburg A, Braun KP, van der Zwan A, Klijn CJ (2014). Recent advances in moyamoya disease: pathophysiology and treatment. Curr Neurol Neurosci Rep.

[6] Yasaka M, Ogata T, Yasumori K, Inoue T, Okada Y (2006). Bottle neck sign of the proximal portion of the internal carotid artery in moyamoya disease. J Ultrasound Med.

[7] Parmar RC, Bavdekar SB, Muranjan MN, Limaye U (2000). Chorea: an unusual presenting feature in pediatric Moyamoya disease. Indian Pediatr.

[8] Thanavaro JL, Nemani N, Price HI (2013). Moyamoya disease: a case of vanishing cerebral vessels. J Am Assoc Nurse Pract.

[9] Ikeda H, Sasaki T, Yoshimoto T, Fukui M, Arinami T (1999). Mapping of a familial moyamoya disease gene to chromosome 3p24.2-p26. Am J Hum Genet.

[10] Yamada H, Deguchi K, Tanigawara T, Takenaka K, Nishimura Y, Shinoda J (1997). Clin Neurol Neurosurg.

[11] Yamauchi T, Tada M, Houkin K, Tanaka T, Nakamura Y, Kuroda S (2000). Linkage of familial moyamoya disease (spontaneous occlusion of the circle of Willis) to chromosome 17q25. Stroke.

[12] Yoshida Y, Yoshimoto T, Shirane R, Sakurai Y (1999). Clinical course, surgical management, and long-term outcome of moyamoya patients with rebleeding after an episode of intracerebral hemorrhage: An extensive follow-Up study. Stroke.

[13] Battistella PA, Carollo C (1997). Clinical and neuroradiological findings of moyamoya disease in Italy. Clin Neurol Neurosurg.

[14] Hallemeier CL, Rich KM, Grubb RL, Chicoine MR, Moran CJ, Cross DT (2006). Clinical features and outcome in North American adults with moyamoya phenomenon. Stroke.

[15] Kraemer M, Heienbrok W, Berlit P (2008). Moyamoya disease in Europeans. Stroke.

[16] Tamogami R, Oi S, Nonaka Y, Miwa T, Abe T (2010). Clinical prognosis and therapeutic aspects in management of pediatric moyamoya disease. Nippon Rinsho.

